# Kidney Function as a Determinant of HDL and Triglyceride Concentrations in the Australian Population

**DOI:** 10.3390/jcm5030035

**Published:** 2016-03-08

**Authors:** Michael Thompson, Udayan Ray, Richard Yu, Andrew Hudspeth, Michael Smillie, Neville Jordan, Janet Bartle

**Affiliations:** Royal Hobart Hospital, University of Tasmania, GPO Box-1061, Hobart 7000, Australia; michael.thompson@ths.tas.gov.au (M.T.); richard.yu@ths.tas.gov.au (R.Y.); andrew.hudspeth@ths.tas.gov.au (A.H.); michael.smillie@ths.tas.gov.au (M.S.); neville.jordan@ths.tas.gov.au (N.J.); janet.bartle@ths.tas.gov.au (J.B.)

**Keywords:** CKD, CVD, HDL, kidney transplantation, lipids, triglycerides

## Abstract

Background: Chronic kidney disease (CKD) is a potent risk factor for cardiovascular disease (CVD). CVD risk increases in a stepwise manner with increasing kidney impairment and is significantly reduced by kidney transplantation, suggesting a causal relationship. Dyslipidemia, a well recognised CVD risk factor, is highly prevalent in CKD. While dyslipidemia is a risk factor for CKD, kidney impairment can also induce a dyslipidemic state that may contribute to the excess burden of CVD in CKD. We utilised a multipronged approach to determine whether a causal relationship exists. Materials and Methods: Retrospective case-control analysis of 816 patients admitted to the Royal Hobart Hospital in 2008–2009 with different degrees of kidney impairment and retrospective before-after cohort analysis of 60 patients who received a transplanted kidney between 1999 and 2009. Results: Decreased estimated GFR (eGFR) was independently associated with decreased high density lipoprotein (HDL, *p* < 0.0001) and increased triglyceride concentrations (*p* < 0.01) in multivariate analysis. There was no significant relationship between eGFR and low density lipoprotein (LDL) or total cholesterol in multivariate analysis. Kidney transplantation increased HDL (*p* < 0.0001) and decreased triglyceride (*p* = 0.007) concentration, whereas there was no significant change in LDL and total cholesterol. These effects were dependent on maintenance of graft function, statin therapy (those who were on) if graft failure occurred then HDL again decreased and triglycerides increased. Conclusions: Kidney transplantation ameliorated alterations in plasma lipoprotein profile associated with kidney impairment, an effect that was dependent on the maintenance of graft function. These data suggest that kidney function is a determinant of HDL and triglyceride concentrations in patients with CKD.

## 1. Introduction

The prevalence of chronic kidney disease (CKD) is increasing in Australia and worldwide [1,2]. Cardiovascular disease (CVD) risk and mortality are significantly elevated in patients with CKD and increase in a stepwise manner as kidney function deteriorates to be greatest in end-stage kidney disease (ESKD) [3]. Kidney transplantation reduces the progression of atherosclerotic disease compared to pre-transplantation rates and kidney transplant recipients enjoy much lower CVD mortality rates than CKD patients who remain on renal replacement therapy [4,5]. These observations suggest a causal relationship between kidney disease and CVD risk and that the increased CVD risk associated with CKD is at least partially reversible.

Dyslipidemia is highly prevalent in patients with CKD and may be the single greatest contributor to CVD risk [6,7]. The prevalence and degree of dyslipidemia increases proportionally to the degree of kidney impairment and may contribute to the observed stepwise increase in CVD risk [6,8]. Dyslipidemia associated with CKD is characterised by low high density lipoprotein (HDL) and raised triglyceride concentrations [6,9]. Low density lipoprotein (LDL) and total cholesterol concentrations may be normal or moderately raised [6,9].

Dyslipidemia is a risk factor for CKD and accelerates its progression [10]. Furthermore, CKD is often associated with other conditions such as diabetes mellitus where dyslipidemia is highly prevalent and phenotypically similar to the dyslipidemia observed in CKD [6,10]. However, there is also evidence that kidney impairment itself is sufficient to induce disordered lipoprotein metabolism and dyslipidemia [9,11]. In this study we wanted to see if kidney transplantation rectifies the adverse effects of kidney impairment to the dyslipidemic profile observed in CKD. To test our hypothesis we utilised a multipronged approach including case-control analysis of patients with different degrees of kidney impairment and retrospective before-after cohort analysis of patients undergoing kidney transplantation to determine whether a causal relationship exists.

## 2. Materials and Methods

### 2.1. Study Design

We conducted retrospective case-control and before-after cohort analysis of patients cared for at the Royal Hobart Hospital. Patients admitted to hospital with ESKD (estimated glomerular filtration rate (eGFR) < 15 mL/min/1.73 m^2^) in 2008–2009 were compared to age- and gender-matched controls with eGFRs of 15–30, 30–60 and >60 mL/min/1.73 m^2^. Age- and gender- matched controls were randomly selected from eligible patients meeting study inclusion criteria admitted to the hospital between 2008 and 2009 using a hospital database. Cohort analysis of patients who received a transplanted kidney between 1999 and 2009 was used to complement the case-control approach. Patients aged between 18 and 90 years were included in the study. Patients were excluded if they developed post-transplantation diabetes mellitus, received multiple organ transplants, had previously received a transplanted organ or had a diagnosed familial hyperlipidemic syndrome. The recruited group of patients who had received statin during CKD period continued on statins in the post transplantation period too. This practice was not shown to make any significant difference in the LDL outcome but showed positive changes in the HDL level following the transplant. The study was approved by the Tasmanian Health and Medical Research Human Ethics Committee. Due to the study’s retrospective nature, the Committee exempted it from the need for informed consent.

### 2.2. Laboratory Methods

Abbott’s CI-8200 main biochemistry analyser was used for routine biochemistry tests including albumin [12,13]. Plasma glucose, urea, creatinine and lipoproteins were measured by the standard spectrophotometric methods [14,15,16,17,18]. LDL values were calculated using the Friedwald’s laboratory formula [14]. The Pathology Laboratory at the Royal Hobart Hospital takes part in National Accreditation and Testing Authority assessment cycle and the External Quality Assessment Program of the Royal College of Pathologists of Australasia.

### 2.3. Data Preparation and Analysis

For case-control analysis, univariate correlations across categories of eGFR were made using a one way ANOVA with post-test analysis for linear trend. Forward stepwise regressions were used for multivariate analysis. For patients who received a kidney transplant, biochemical data were divided into pre- and post-transplantation sets. Comparisons between pre- and post-transplant values were made using a paired *t*-test. Data that were not normally distributed were log transformed. A two-sided P value of less than 0.05 (*p* < 0.05) was considered statistically significant. P values were corrected using the Student Newman-Keuls method for multiple comparisons between non-orthogonal groups. All data were analysed in SigmaStat version 3.5™ and graphs were compiled in SigmaPlot version 10™. Results were expressed as mean ± standard deviation unless otherwise stated.

## 3. Results

### 3.1. Patient Characteristics

Of the 204 patients with ESKD who met study criteria, 120 were male and 84 female. Mean age was 65.1 years. Additional sociodemographic, clinical and laboratory characteristics of the case-control study population are shown in Table 1. Sixty kidney transplant recipients met the criteria for inclusion in the study (Table 2). For these patients the aetiology of ESKD was glomerulonephritis [15], hypertension [13], type 1 diabetes mellitus [9], adult polycystic kidney disease [8], reflux nephropathy [5], type 2 diabetes mellitus [4], renal canaliculi and obstructive nephropathy [2], ANCA+ vasculitis [1], Prune Belly Syndrome [1], chronic interstitial inflammation [1] and acute tubular necrosis [1]. All patients receiving a kidney transplant had been on dialysis for a period of months to years prior to transplantation. Following kidney transplantation the most frequently used immunosuppression regime in our institute consists of mycophenolate mofetil, prednisolone and tacrolimus or cyclosporine (Table 2). In addition to immunosuppressant medications, 95% of kidney transplant recipients were taking lipid-lowering statin drugs. All patients taking statins commenced treatment at least 6 months prior to transplantation.

### 3.2. Effect of Kidney Function on Plasma Lipoprotein Concentrations

Increasing kidney impairment was associated with decreasing concentrations of total cholesterol, LDL and HDL and an increasing triglyceride concentration (all *p* < 0.0001) in univariate analysis. Albumin decreased with increasing kidney impairment (*p* = 0.006). After adjustment for lipid lowering therapy, the associations between kidney function and total cholesterol (*r* = −0.034) and LDL (*r* = −0.063) were not statistically significant, whereas the relationship between kidney function and HDL (*r* = −0.292, *p* < 0.0001) and triglycerides (*r* = 0.246, *p* < 0.001) remained significant. The relationship between kidney function, HDL (*r* = −0.234, *p* < 0.001) and triglycerides (*r* = 0.151, *p* < 0.01) remained significant after adjustment for a history of hypertension, diabetes mellitus, smoking status and albumin concentration.

**Figure 1:**
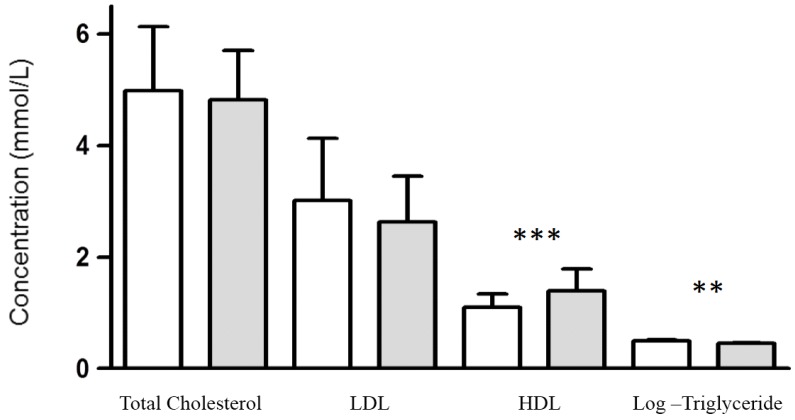
Paired before-after plasma lipoprotein concentration for patients who received a kidney transplant. Values are displayed as mean ± standard deviation. To convert values HDL to mg/dL, multiply by 38.67. To convert values for triglycerides to mg/dL, multiply by 88.57. ** *p* = 0.007. *** *p* < 0.0001. Abbreviations: HDL, high density lipoprotein; LDL, low density lipoprotein.

**Figure 2:**
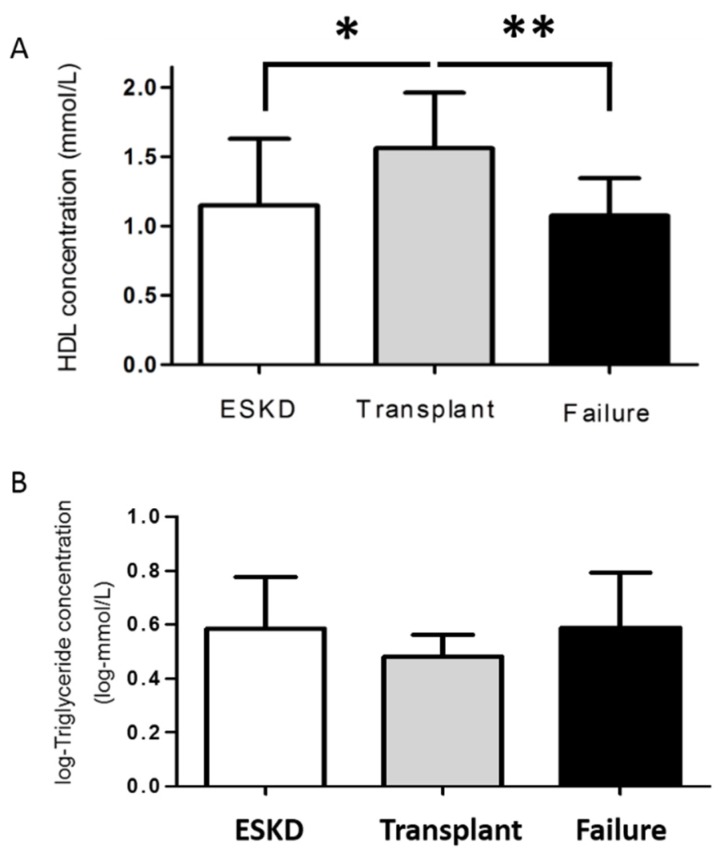
Average HDL (**A**) and triglyceride (**B**) concentrations according to kidney function for patients that experienced graft failure. Value are displayed as mean ± standard deviation. To convert values HDL to mg/dL, multiply by 38.67. To convert values for triglycerides to mg/dL, multiply by 88.57. * *p* = 0.001. ** *p* < 0.01. Abbreviations: HDL, high density lipoprotein.

Kidney transplantation resulted in a significant increase in eGFR (*p* < 0.0001, data not shown). To investigate the effect kidney transplantation on plasma lipoprotein profile paired before-after lipoprotein concentrations were compared (Figure 1). Following restoration of kidney function, there was a significant increase in HDL (*p* < 0.0001) and decrease in triglyceride (*p* = 0.007) concentrations (Figure 1). Total cholesterol (*p* = 0.33), LDL (*p* = 0.06) and albumin (*p* = 0.89) did not change significantly following kidney transplantation (Figure 1). These changes in lipoprotein concentration were dependent on graft function being maintained. If graft function was not maintained, such as in the case of graft failure (*n* = 4), then HDL again decreased (*p* < 0.01, Figure 2). While the effect of graft failure on triglyceride concentration did not reach statistical significance, there was a suggestive increase (Figure 2), which may reach significance in larger cohorts.

**Figure 3:**
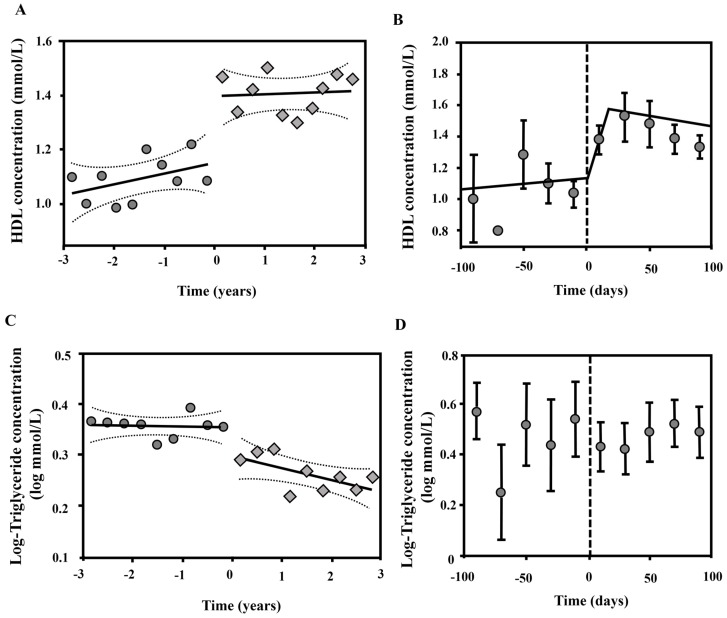
HDL and triglyceride concentration with respect to time prior to and following kidney transplantation. (**A**) Linear regression analysis of HDL concentration in the 3 years prior to and following kidney transplantation; (**B**) Piecewise 3 segment linear regression of HDL concentration in the 100 days prior to and following kidney transplantation; (**C**) Linear regression analysis of log triglyceride concentration in the 3 years prior to and following kidney transplantation; (**D**) Log triglyceride concentration in the 100 days prior to and following kidney transplantation. Symbols represent mean values for all observations within 4 month time intervals (**A**, **C**) or 20 day time intervals (**B**, **D**) represent the standard deviation for all observations within a period. To convert values HDL to mg/dL, multiply by 38.67. To convert values for triglycerides to mg/dL, multiply by 88.57. Abbreviations: HDL, high density lipoprotein.

Linear regression analysis demonstrated no significant change in HDL in the three years preceding or following kidney transplantation (Figure 3A). However, there was a significant difference between the mean HDL concentration before and after kidney transplantation (*p* < 0.001, Figure 3A). To determine the kinetics HDL concentration in the peri-transplant period, HDL measurements from 100 days either side of kidney transplantation were analysed using a Piecewise 3 Segment Linear Regression. This demonstrated a rapid, significant (*r* = 0.649, *p* < 0.0001) increase following kidney transplantation (Figure 3B). There was no significant change in HDL concentration in the 100 days preceding or days 20–100 following transplantation (Figure 3B). Linear regression analysis of triglyceride concentration demonstrated no significant change in the three years preceding kidney transplantation (Figure 3C). Following kidney transplantation, the mean triglyceride concentration was significantly lower (*p* < 0.001) and there was a gradual, but significant (*r* = −0.188, *p* = 0.0008) decrease in triglyceride concentration over a period of years (Figure 3C). Analysis of triglyceride kinetics in the peri-transplant period demonstrated no acute change in triglyceride concentration in the 100 days before or after kidney transplantation (Figure 3D).

## 4. Discussion

CKD is often associated with lipid abnormalities, particularly low HDL and raised triglycerides [6,9]*.* In this study, we observed similar relationships between kidney impairment, HDL and triglyceride concentrations. To our knowledge, this is the first time such relationships have been described in the Australian population. The relationship between kidney function, HDL and triglycerides remained significant even after adjustment for a wide variety of influencing factors, such as the presence of lipid lowering therapy, diabetes mellitus and smoking status. In contrast, the negative correlation between kidney function, LDL and total cholesterol observed in univariate analysis was entirely accounted for by the increasing use of statins and not significant in multivariate analysis. These data support the hypothesis that CKD-associated CVD risk may be partially mediated by the increasingly prevalence and severity of pro-atherogenic dyslipidemia as kidney function deteriorates.

Kidney transplantation coincided with significant alterations in HDL and triglyceride concentrations that ameliorated the dyslipidemic profile seen in ESKD. Analysis of HDL concentrations in time demonstrated that HDL increased significantly following transplantation. This time period corresponds closely to the half-life for HDL biosynthesis [19], suggesting a rapid effect of kidney transplantation on HDL metabolism. Excepting this sudden, significant increase in HDL following kidney transplantation, HDL concentrations were relatively stable, with no significant alteration observed in the three year period preceding or following kidney transplantation. These temporal kinetics implicate kidney transplantation as a major influence on HDL concentration in these patients and suggest a rapid and lasting effect of kidney transplantation on HDL concentration. In contrast to HDL, the decrease triglyceride concentration following kidney transplantation occurred gradually over a period of years with no acute change observed. This may be due to the induction of immunosuppressive therapy, which may have masked acute changes in triglyceride concentration, and gradual tapering of prednisolone dosage over the months to years following transplantation [20,21]. Further supporting the hypothesis that kidney function is a determinant of lipoprotein profile, changes in HDL and triglycerides following kidney transplantation were dependent on the successful engraftment and maintenance of graft function. If graft function was not maintained then HDL concentration once again decreased and triglyceride concentration increased. Taken together, these observations suggest that kidney function is an important determinant of HDL and, potentially, triglyceride concentrations in patients with CKD and kidney transplant recipients.

Hypoalbuminemia may contribute to dysregulation of lipoprotein metabolism, particularly HDL, and consequent dyslipidemia in CKD [22]*.* Albumin has an important role in transporting cholesterol to HDL and thus hypoalbuminemia may contribute to the high prevalence of low HDL observed in patients with CKD [23]. We found that HDL and triglycerides remained significantly associated with kidney function in multivariate analysis that included albumin concentration. Additionally, there was no significant change in albumin following kidney transplantation, suggesting that the effect of kidney function on HDL and triglyceride concentrations was not due to a quantitative change in albumin in our patient population.

Several limitations should be considered in interpreting the results presented in this study. First, immunosuppressive medications such a prednisolone may have significant, but presently unavoidable, effects on lipoprotein metabolism and concentration [20,21]. Similarly, interpretation of the impact of kidney function and kidney transplantation on LDL and total cholesterol was confounded by the frequent use of statin drugs in our patient population. Patients with increasing kidney impairment are a well recognised, high risk population for CVD and consequently often placed on statin therapy, a bias well demonstrated in our population. Furthermore, while we have demonstrated quantitative improvements in HDL and triglyceride concentrations following kidney transplantation, the functional properties of these lipoproteins has not been examined. Increasing evidence suggests that the functional properties of plasma lipoproteins, particularly HDL, are more robust indicators of CVD risk that the absolute amount [24,25]. Determining the effect of kidney transplantation on functional integrity of HDL will be important to future strategies aiming to utilise this promising therapeutic target.

In summary, our study utilised a powerful, multipronged case-control and before-after cohort approach to determine whether a causal relationship between kidney function and plasma lipoprotein profile exists. Our data suggest that kidney function is a determinant of HDL and triglyceride concentrations in Australian patients with CKD. These results support the hypothesis that CKD-associated CVD risk may be partially mediated through the effect of kidney function on HDL and triglyceride concentrations. Reduction in long-term CVD mortality in kidney transplant recipients may further be due to improvements in lipoprotein profile following kidney transplantation.

## Figures and Tables

**Table 1:** Characteristics of 204 patients with ESKD compared to age- and gender-matched controls with eGFRs of >60, 30–60 and 15–30 mL/min/1.73 m^2^.

Risk Factor	eGFR (mL/min/1.73 m^2)^				*p* Trend
	>60	30–60	15–30	<15	
Diabetes mellitus (%)	21.6	37.6	35.8	32.8	ns
Hypertension (%)	42.1	70.5	69.6	69.1	<0.0001
Smoking					
Current (%)	10.8	22.0	8.8	15.1	ns
Former (%)	17.2	44.4	33.3	21.1	ns
Total cholesterol (mmol/L)	4.99 ± 1.36	4.96 ± 2.04	4.36 ± 1.46	4.26 ± 1.10	<0.0001
LDL (mmol/L)	2.99 ± 1.07	2.88 ± 1.48	2.42 ± 1.28	2.33 ± 0.87	<0.0001
HDL (mmol/L)	1.21 ± 0.39	1.10 ± 0.37	1.01 ± 0.37	1.02 ± 0.28	<0.0001
log-Triglycerides (log-mmol/L)	0.40 ± 0.14	0.43 ± 0.14	0.47 ± 0.14	0.48 ± 0.12	<0.0001
Serum albumin (g/L)	35.6 ± 6.0	34.9 ± 6.9	32.6 ± 7.8	33.5 ± 4.5	0.006
Medication use					
Statin (%)	27.5	33.8	46.1	81.3	<0.0001
Fibrate (%)	2.0	2.5	2.9	2.0	ns
Ezetimibe (%)	0.9	3.4	1.5	2.0	ns

Values are displayed as mean ± standard deviation. To convert values for LDL, HDL, and total cholesterol to mg/dL, multiply by 38.67. To convert values for triglycerides to mg/dL, multiply by 88.57. Abbreviations: ESKD, end stage kidney disease; eGFR, estimated glomerular filtration rate; ns, not significant; HDL, high density lipoprotein; LDL, low density lipoprotein.

**Table 2:** Sociodemographic, laboratory and clinical characteristics of 60 kidney transplant recipients.

Age (years)	51.1
Gender (M/F)	36/24
Hypertension (%)	95.0
Diabetes mellitus (%)	33.3
Smoking	
Former (%)	20.0
Current (%)	8.3
Lipid lowering	
Statin (%)	95.0
Fibrates (%)	0.0
Ezetimibe (%)	1.6
Immunosuppressant	
Predniosolone (%)	98.3
Mycophenolate (%)	96.7
Tacrolimus (%)	81.7
Cyclosporin (%)	11.7
Sirolimus (%)	4.0
Azathiopurine (%)	4.0
Lefluonamide (%)	2.0

Abbreviations: M, male; F, female; %, percentage.

## Abbreviations

The following abbreviations are used in this manuscript:

CKD	Chronic kidney disease
CVD	Cardiovascular disease
eGFR	Estimated glomerular filtration rate
ESKD	End stage kidney disease
HDL	High density lipoprotein
LDL	Low density lipoprotein
